# The Use of the SMART-COP Score in Predicting Severity Outcomes Among Patients With Community-Acquired Pneumonia: A Meta-Analysis

**DOI:** 10.7759/cureus.27248

**Published:** 2022-07-25

**Authors:** Rahat A Memon, Muhammad Affan Rashid, Sahithi Avva, Venkata Anirudh Chunchu, Huda Ijaz, Zubair Ahmad Ganaie, Albeena Kabir Dar, Neelum Ali

**Affiliations:** 1 Internal Medicine, California Institute of Behavioural Neurosciences and Psychology, Fairfield, USA; 2 Medicine, Allama Iqbal Medical College, Lahore, PAK; 3 Medicine, Lady Hardinge Medical College, New Delhi, IND; 4 Medicine, Avalon University School of Medicine, Willemstad, CUW; 5 Medical College, Allama Iqbal Medical College, Lahore, PAK; 6 Internal Medicine, Holy Family Red Crescent Medical College, Srinagar, IND; 7 Internal Medicine, Holy Family Red Crescent Medical College Hospital, Dhaka, BGD; 8 Internal Medicine, University of Health Sciences, Lahore, PAK

**Keywords:** diagnostic test accuracy, prognosis, severity, community-acquired pneumonia (cap), smart-cop

## Abstract

Pneumonia is a pathological process of interstitial lung tissue and distal airway and alveolar infection and infiltration. SMART-COP (systolic blood pressure, multilobar infiltrates, albumin, respiratory rate, tachycardia, confusion, oxygen, and pH) is a severity score method designed to identify individuals who require intensive respiratory or vasopressor support (IRVS) support due to pneumonia. Therefore, it is important for management decisions in pneumonia. This meta-analysis was conducted to determine the performance of the SMART-COP score in predicting the prognosis and severity of patients presenting with community-acquired pneumonia (CAP). The current meta-analysis was performed using Preferred Reporting Items for Systematic Reviews and Meta-Analyses (PRISMA) guidelines. A systematic search was conducted using Medline, Embase, and CINAHL to identify relevant studies assessing the validity of the SMART-COP score in predicting the severity of patients with CAP. Overall, nine studies were included in the current meta-analysis. A pooled sensitivity of the SMART-COP score to predict the use of IRVS is 89% (95% CI: 84%-92%) while its specificity is 68% (95% CI: 65%-70%). The pooled sensitivity of the SMART-COP score to predict 30-day mortality is 92% (95% CI: 89%-94%) while its specificity is 39% (95% CI: 37%-42%). To summarize, SMART-COP is a new, eight-variable instrument that appears to accurately identify patients with CAP who will require IRVS and 30-day mortality. Our findings show that SMART-COP will be a valuable tool for clinicians in accurately predicting illness severity in CAP patients as compared to other scoring systems. SMART-COP can be useful to identify patients who need urgent management.

## Introduction and background

Pneumonia is a pathological process of interstitial lung tissue and distal airway and alveolar infection and infiltration [1]. Its clinical definition is a group of symptoms, including tachypnea, increased sputum production, productive cough, chills, increased bronchial lung sounds, fever, or pleuritic chest discomfort, all of which are followed by chest X-ray infiltration (CXR) [2]. The incidence of pneumonia is 20% to 30% in low- and middle-income countries while in developed countries, its incidence is 3% to 4% [3-4]. It is one of the most common causes of mortality and morbidity. Based on studies, it is one of the top five causes of death in old age people. Patients who require hospital admission had the highest morbidity rates from community-acquired pneumonia (CAP), with a 30-day mortality rate of up to 13% recorded in those patients [5-6].

Even though most patients have mild symptoms, 5% present with shock, multiorgan dysfunction, or hypoxaemic respiratory failure [7]. Identification of patients who will require advanced support or who are at risk of poor prognosis is important. Several assessment tools have been developed and validated to guide physicians in managing patients with CAP. CURB-65 (confusion, uremia, respiratory rate, BP, age > 65 years) and pneumonia severity index (PSI) are two popular tools [8-9]. SMART-COP (systolic blood pressure, multilobar infiltrates, albumin, respiratory rate, tachycardia, confusion, oxygen, and pH), developed by a group of Australian academics, is one of the most recent methods for assessing pneumonia. SMART-COP is a severity score method designed to identify individuals who require intensive respiratory or vasopressor support (IRVS) support and intensive care unit (ICU) admission due to pneumonia. The SMART-COP score includes tachycardia, systolic blood pressure (SBP), oxygen saturation (SpO2), potential hydrogen (pH), and acute confusion [10]. In comparison to previous scoring systems, this score is more sensitive and specific in identifying patients at risk of severe disease and predicting the requirement for ICU care based on the likelihood of requiring intense respiratory or vasopressor support [2].

As compared to other scoring systems, SMART-COP can be useful in identifying or recognizing patients with increased risk for a severe form of the disease [2]. Therefore, it is important for management decisions in pneumonia. It is different from CURB-65 and PSI in that an important goal of these tools is the identification of seriously ill patients who need to be referred to ICU while the SMART-COP score has greater effectiveness in identifying patients who require IRVS support [10]. Because ICU admission criteria differ between regions, the current study focused on features associated with vasopressor support or intensive respiratory instead of simple ICU admission because these are more likely to be objective markers of CAP severity across institutions and health care systems.

The use of the SMART-COP score in pneumonia patients to assist Emergency Physicians in determining disease severity and predicting the need for early intensive respiratory or vasopressor support. It will reduce their ED length of stay by allowing for an earlier decision of disposition, resulting in more effective and efficient use of resources in developing countries. Therefore, this meta-analysis was conducted to determine the performance of the SMART-COP score in predicting the prognosis and severity of patients presenting with CAP.

## Review

Methodology

The current meta-analysis was performed using Preferred Reporting Items for Systematic Reviews and Meta-Analyses (PRISMA) guidelines.

Search Strategy

A systematic search was conducted using Medline (PubMed), Embase, and CINAHL to identify relevant studies assessing the validity of the SMART-COP score in predicting the severity of patients with CAP. Citation searching was also done on Web of Science, Scopus, and Google Scholar to maximize sensitivity. Key terms used to search relevant articles included “SMART-COP”, “validity”, “community-acquired pneumonia”, “IRVS”, and “severity outcomes”. Boolean operators (AND, OR) were also used while searching for relevant articles. The search was performed for articles in English, and no restrictions were placed on the year of publication. Reference lists of retrieved articles were also screened to make sure a comprehensive search.

Eligibility Criteria

Eligible studies were retrospective or prospective, assessing the validity of the SMART-COP score in predicting IRVS use and 30-day mortality. The evaluation had to be done within 24 hours of hospital admission. Studies assessing the validity of SMART-COP scores in children were excluded from the current meta-analysis. Two investigators independently evaluated all studies. Non-relevant studies were excluded based on the study title and abstract. The full text was obtained of all relevant articles, and two investigators independently assessed the eligibility criteria and extracted the data from articles based on first author name, year of publication, study type, outcomes, study setting, mean age, and inclusion criteria. Any disagreement that occurred between investigators was resolved through discussion or the involvement of a third investigator.

Quality Assessment

The quality of studies was assessed by two authors independently utilizing the revised Quality Assessment of Diagnostic Accuracy Studies (QUADAS-2) tool. This tool assessed the risk of bias about applicability by assessing four important domains that included the flow of patients, reference standard, index text, and patient selection through the study and timing of tests.

Data Abstraction

The primary outcome of interest was IRVS usage while secondary outcomes included admission to the ICU and 30-day mortality, which was counted per event and defined as reported in the included articles. Diagnostic accuracy measures were recorded, such as the area under the curve (AUC), specificity, sensitivity, negative predictive values (NPV), and positive predictive values (PPV), to perform a diagnostic meta-analysis.

Statistical Analysis

Data analysis was done using STATA version 16.0 (metadata and metan packages; Stata Statistical Software: Release 16. College Station, TX: StataCorp LLC) and review manager (RevMan) software (version 5.4.1; Review Manager (RevMan) [Computer program]. The Cochrane Collaboration, 2020). The pooled sensitivity, specificity, and negative and positive likelihood ratios, along with their 95% confidence intervals, were obtained using a bivariate random-effects model. Because several specificities and sensitivities were reported in the articles at different cut-offs, a linear mixed model was utilized with a correlation structure in order to take the dependence of the measures into account. Forest plots of the specificity and sensitivity were utilized for the graphical representation of the results. The I-square index was used to measure heterogeneity, the Cochran test was used for testing heterogeneity, and a p-value less than 0.05 was considered statistically significant.

Results

**Figure 1:**
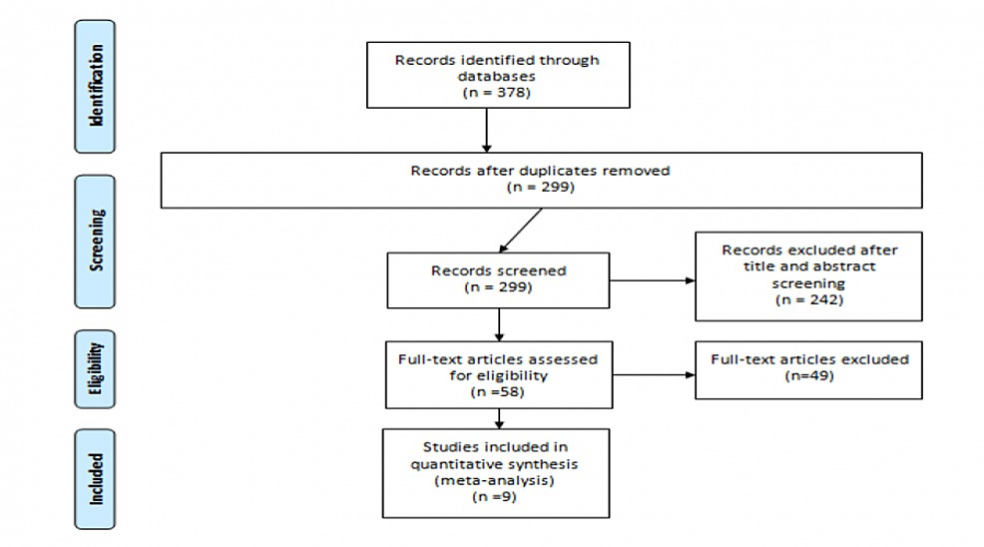
PRISMA flow diagram showing the selection of articles for review PRISMA: Preferred Reporting Items for Systematic Reviews and Meta-Analyses

The PRISMA flow chart presenting the articles selected for this meta-analysis is shown in Figure 1. Through a systematic search of Medline, Embase, and CINAHL, 378 articles were retrieved, among which 79 were duplicates. Of the 299 articles, 242 articles were excluded based on abstract and title. The full text of 58 articles was obtained and reviewed for eligibility criteria. Overall, eight articles fulfilled the eligibility criteria. One article was identified by manual searching of the references. Overall, nine articles were included in the current meta-analysis. Table 1 shows the characteristics of the included studies. Among all included studies, eight were prospective [2,10-16] while one was conducted retrospectively [17]. Most of the included studies used three as a cut-off of the SMART-COP score to predict IRVS need and 30-day mortality in patients [10-15,17].

**Table 1:** Characteristics of included studies CAP: community-acquired pneumonia; IRVS: intensive respiratory or vasopressor support

Author	Year of Publication	Study Design	Outcomes	Sample Size	Cut-off of SMART-COP Score	Mean Age	Inclusion Criteria
Alici et al [11]	2015	Prospective	Need of IRVS	84	More than or equal to 3	58.6 Years	-
Chalmer et al [12]	2008	Prospective	Need of IRVS	335	More than or equal to 3	<50 years	Patients less than 50 years of age and presenting with a new infiltrate on a chest radiograph.
Charles et al [10]	2008	Prospective	Need of IRVS, 30-day mortality	862	More than or equal to 3	-	Age of at least 18 years, at least 1 symptom of CAP, CXR changes
								Davis et al [13]	2010	Prospective	Need of IRVS	184	More than or equal to 3	50.1 Years	Adult patients with sepsis
Ehsanpoor et al [2]	2019	Prospective	Need of IRVS, 30-day mortality	143	More than or equal to 5	68.13 Years	Patients with age older than 18 years old, having at least 3 specific clinical presentations and signs of pneumonia
								Fukuyama et al [14]	2011	Prospective	30-day mortality	298	More than or equal to 3	76 Years	Patients with age greater than or equal to 18 patients and admitted to the hospital with pneumonia
Hamza et al [15]	2019	Prospective	30-day mortality	76	More than or equal to 3	59.32 Years	Patients with age greater than or equal to 18 patients and admitted to the hospital with pneumonia
Masuduzzaman et al [16]	2020	Prospective	30-day mortality	54	More than or equal to 4	46.74 Years	Patients with age greater than or equal to 18 patients and admitted to the hospital with pneumonia
Williams et al [17]	2018	Retrospective	30-day mortality	618	More than or equal to 3	56 Years	Patients with age greater than or equal to 18 patients and admitted to the hospital with pneumonia

Risk of Bias

**Figure 2:**
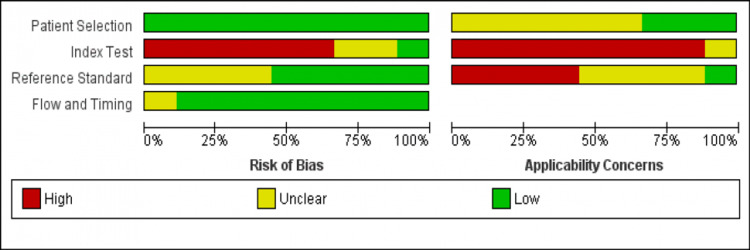
Quality assessment

The results of quality assessment using the QUADAS-2 tool are represented in Figure 2. Overall, the applicability of included studies was moderate. All included studies did not use a pre-assigned index test cut-off.

Predictive Value of the SMART-COP Score to Predict the Need for IRVS

**Figure 3:**
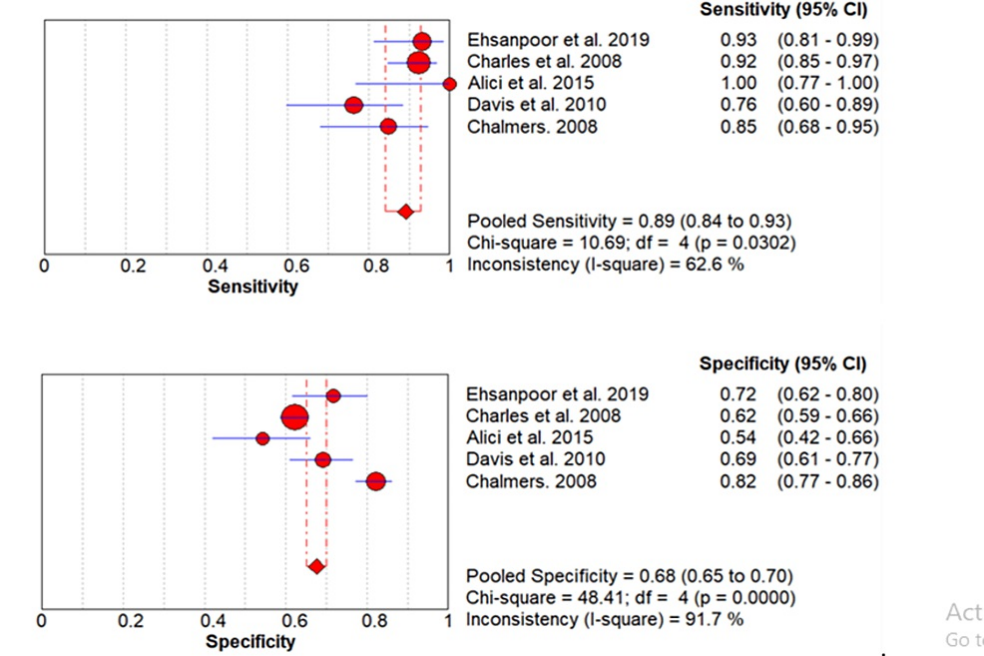
Pooled sensitivity and specificity for IRVS Values are shown with 95% confidence intervals. Red circles are showing the sensitivity and specificity of individual studies while diamonds are showing pooled sensitivity and specificity. Lines are showing a 95% confidence interval. IRVS: intensive respiratory or vasopressor support Sources: [2,10-13]

The rate of IRVS use in the included studies ranged from 6.38% to 65.48% [2,10-13] as shown in Table 2, and the pooled incidence of IRVS use for the five articles was 12% (95% CI: 11%-14%). There was significant heterogeneity (p-value=0.001). A meta-analysis of the predictive value of SMART-COP score for IRVS use was performed by including five studies. A pooled sensitivity of the SMART-COP score to predict the use of IRVS is 89% (95% CI: 84%-93%) while its specificity is 68% (95% CI: 65%-70%) as shown in Table 3. The forest plot is shown in Figure 3. The pooled AUC is 0.84.

**Table 2:** Rate of IRVS need and 30-day mortality in included studies IRVS: intensive respiratory or vasopressor support

Studies	IRVS (%)	Mortality (%)
Alici et al, 2015 [11]	65.48	7.14
Chalmers et al, 2008 [12]	16.42	-
Charles et al, 2008 [10]	6.38	-
Davis et al, 2010 [13]	29.89	-
Ehsanpoor et al, 2019 [2]	38.46	20.28
Fukuyama et al, 2011 [14]	-	10.07
Hamza et al, 2019 [15]	-	22.37
Masuduzzaman et al, 2020 [16]	-	5.56
Williams et al, 2021 [17]	-	23.48

**Table 3:** Meta-analysis of predictive data for IRVS and 30-day mortality Presented with a 95% confidence interval IRVS: intensive respiratory or vasopressor support

Measures	Outcomes	
	IRVS need	30-day mortality
Pooled Sensitivity	89 (84-93)	92 (89-94)
Pooled Specificity	68 (65-70)	39 (37-42)
Pooled positive LR	4.14 (2.68-6.39)	1.95 (1.56-2.44)
Pooled negative LR	0.39 (0.31-0.49)	0.07 (0.02-0.20)
Pooled AUC	0.84 (0.79-0.89)	0.51 (0.44-0.57)
Diagnostic odds ratio	17.89 (9.67-33.09)	15.89 (4.67-54.02)

Predictive Value of the SMART-COP Score to Predict 30-Day Mortality

**Figure 4:**
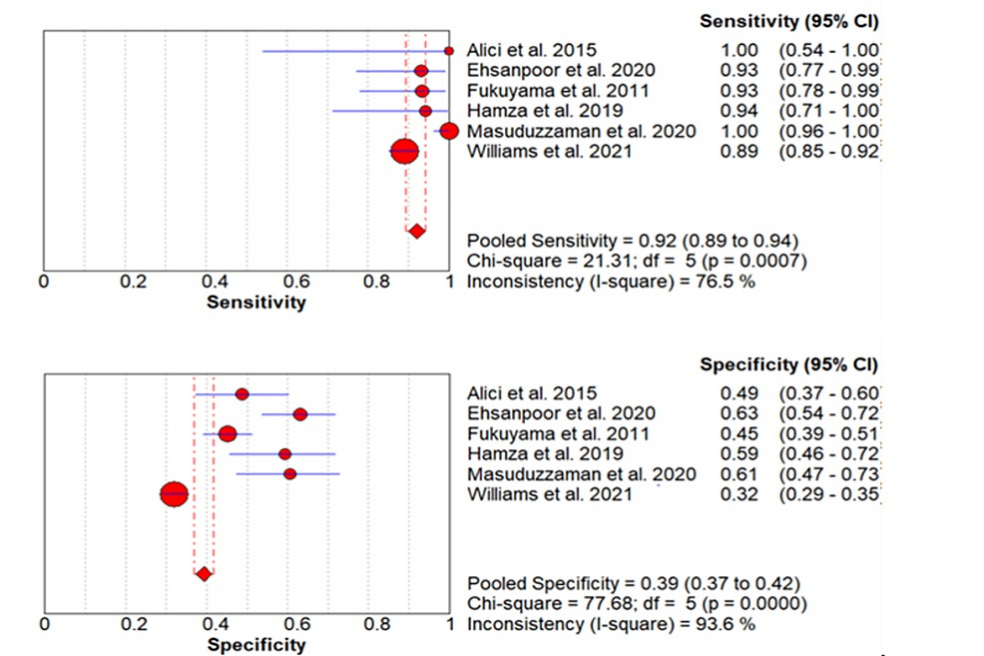
Pooled sensitivity and specificity for 30-day mortality Values are shown with 95% confidence intervals Red circles are showing the sensitivity and specificity of individual studies while diamonds are showing pooled sensitivity and specificity. Lines are showing a 95% confidence interval. IRVS: intensive respiratory or vasopressor support Sources: References [2,11,14-17]

The incidence of 30-day mortality in the included studies ranged from 5.56% to 23.48% [2,11,14-17] as shown in Table 2. The pooled incidence of 30-day mortality for the six studies was 18% (95% CI: 16%-19%). A meta-analysis of the predictive value of the SMART-COP score for 30-day mortality was performed by including six studies. The pooled sensitivity of the SMART-COP score to predict 30-day mortality is 92% (95% CI: 89%-94%) while its specificity is 39% (95% CI: 37%-42%) as shown in Table 3. The forest plot is shown in Figure 4. The pooled AUC is 0.51.

Discussion

Current assessment tools to predict pneumonia severity, like CURB-65 and PSI, are used to predict ICU admission and 30-day mortality among pneumonia patients. However, this outcome is based on the comorbid illness and age of the patients. Therefore, these tools cannot be used to predict the need for IRVS among patients with CAP [18]. In reality, clinicians may use such characteristics to determine whether a patient's situation may be classified as NFR, meaning it is not fit for vigorous medical treatment. It was identified from the current meta-analysis that the sensitivity of the SMART-COP score to predict IRVS need and 30-day mortality is 89% and 92%, respectively. The current meta-analysis also found that increasing the SMART-COP score was associated with an increased likelihood of IRVS need and 30-day mortality.

A meta-analysis was conducted by Marti et al. to compare various scoring systems in determining the prognosis of pneumonia. The study concluded that new severity scores of CAP in predicting ICU admission and need for IRVS, including SMART-COP score, SCAP (severe community-acquired pneumonia) score, and ATS/IDSA (American Thoracic Society/Infectious Diseases Society of America) 2007 minor criteria, had better discrimination performance as compared to the CURB-65 and PSI [19]. The optimal cut-off for the SMART-COP score for predicting the need for IRVS has varied in different studies [2,10-17]. Certain studies have identified optimal cut-off based on ROC curves and the Youden index, but the values range anywhere from 3 to 5, with slight variation in specificity and sensitivity.

We noticed that the present scores did not include any acute-phase inflammatory markers, but preliminary data suggested that certain markers, such as procalcitonin, could improve the risk validity and strength of the score, hence boosting its value in predicting disease prognosis [20]. Researchers are expected to assess topographies specifically associated with receiving intensive respiratory support (i.e., mechanical ventilation (invasive or noninvasive) or vasopressors therapy to support blood pressure), not just ICU admission, because the principles for ICU admission differ among hospitals, ICUs, and countries [21]. These factors are expected to play a role in determining the severity of CAP in hospitals and care facilities.

While clinical judgment is the most important factor in predicting the severity of pneumonia, clinicians can use high-sensitivity scoring systems for pneumonia risk stratification to know about patients who may need closer monitoring or more aggressive treatment [14]. In the current meta-analysis, it was discovered that SMART-COP was effective in predicting the need for IRVS in our sample. The patient population was diverse, with the majority of them having numerous serious underlying conditions such as diabetes, renal, hepatic, or cardiac failure, obstructive lung disease, and so on. We can use SMART-COP with confidence to identify patients who are at high risk of severe pneumonia and require immediate treatment.

The current meta-analysis has certain limitations. First, studies included in this meta-analysis used different cut-offs, as only two studies used a cut-off of 4 and 5 while other studies used a cut-off of 3 to predict the outcomes of CAP. As only two studies used different cut-offs, we were not able to perform a sub-group analysis. In the future, more studies need to be conducted prospectively, including a larger sample size to determine the cut-off applicable to the general population with CAP. In addition, owing to the differences in study design, methodology, and patient population, this meta-analysis is limited by the heterogeneity of the included studies

## Conclusions

In the end, the current study found that the pooled sensitivity of the SMART-COP score to predict the need for IRVS and 30-day mortality is 89% and 92%, respectively. To summarize, SMART-COP is a new, eight-variable instrument that appears to accurately identify patients with CAP who will require IRVS. Our findings show that SMART-COP will be a valuable tool for clinicians in accurately predicting illness severity in CAP patients. Predicting outcomes in CAP is a major safety concern, and physicians can intervene correctly and quickly by using severity risk categorization.
